# Exploring Attention in Depth: Event-Related and Steady-State Visual Evoked Potentials During Attentional Shifts Between Depth Planes in a Novel Stimulation Setup

**DOI:** 10.3390/vision9020028

**Published:** 2025-04-03

**Authors:** Jonas Jänig, Norman Forschack, Christopher Gundlach, Matthias M. Müller

**Affiliations:** Experimental Psychology and Methods, Wilhelm Wundt Institute for Psychology, Leipzig University, 04109 Leipzig, Germany; jonas.jaenig@uni-leipzig.de (J.J.); norman.forschack@uni-leipzig.de (N.F.); christopher.gundlach@uni-leipzig.de (C.G.)

**Keywords:** visual attention, spatial attention, attentional shifts, depth, 3D, stereoscopic 3D, EEG, ERP, SSVEP

## Abstract

Visuo-spatial attention acts as a filter for the flood of visual information. Until recently, experimental research in this area focused on neural dynamics of shifting attention in 2D space, leaving attentional shifts in depth less explored. In this study, twenty-three participants were cued to attend to one of two overlapping random-dot kinematograms (RDKs) in different stereoscopic depths in a novel experimental setup. These RDKs flickered at two different frequencies to evoke Steady-State Visual Evoked Potentials (SSVEPs), a neural signature of early visual stimulus processing. Subjects were instructed to detect coherent motion events in the to-be-attended-to plane/RDK. Behavioral data showed that subjects were able to perform the task and selectively respond to events at the cued depth. Event-Related Potentials (ERPs) elicited by these events—namely the Selection Negativity (SN) and the P3b—showed greater amplitudes for coherent motion events in the to-be-attended-to compared to the to-be-ignored plane/RDK, indicating that attention was shifted accordingly. Although our new experimental setting reliably evoked SSVEPs, SSVEP amplitude time courses did not differ between the to-be-attended-to and to-be-ignored stimuli. These results suggest that early visual areas may not optimally represent depth-selective attention, which might rely more on higher processing stages, as suggested by the ERP results.

## 1. Introduction

Visuo-spatial attention filters relevant stimuli from the continuous flood of visual information. Implicitly, this filtering function is assumed to operate in three-dimensional (3D) environments. However, the majority of experimental studies on attentional filtering have focused on two-dimensional (2D) environments [1,2,3], neglecting that central aspects of 3D scene analysis like eye vergence control or disparity processing might afford different attentional processes [4,5]. Both in light of ecological validity and concerning the rise of virtual and augmented reality devices, this lack of research limits our understanding of how attention operates in depth-related processing. Consequently, the functional mechanism of attentional shifts along the mid-sagittal axis (i.e., in the depth dimension) has not yet been fully understood and it remains an open question whether neural mechanisms underlying spatial attentional selection in 2D and 3D are comparable.

Different approaches have been pursued to investigate mechanisms of perception in 3D. By means of single-cell recordings, brain areas involved in stereoscopic vision have been identified in non-human primates. The earliest cortical cells sensitive to stereoscopic input are simple and complex cells found in the primary visual cortex (V1). These cells are tuned to a certain disparity, i.e., the offset in the position of the perceived stimulus between retinae [6]. Beyond that, binocular disparity cells have also been found in many other cortical areas of the visual system, including the secondary and tertiary visual cortex (V2 and V3), the medial superior temporal area (MST), and the middle temporal area (MT/V5) [7]. Functional magnetic resonance imaging (fMRI) studies in humans have measured increased activity during 3D tasks in V1 and V2 [8], V3 [9,10], and hMT+/V5 [8], showing that most of the non-human primate findings can be generalized to humans.

While these foundational studies provide a spatially detailed account of the cortical structures contributing to depth perception, psychophysical and electroencephalographic studies have investigated the functional allocation of attentional resources to stimuli presented at different depths at a rudimentary level.

In an exemplary behavioral study, Theeuwes et al. [11] instructed participants to search for a red bar within a stimulus array of green bars and report the tilt of the red bar by pressing a left vs. right button. Half of the stimuli were presented in front, and half of them were presented behind the monitor plane. A rectangle enclosing the stimulation area of one plane preceded the search display and indicated the target position with 80% validity. Participants showed slower reaction times and more errors in invalidly cued trials, providing evidence for the hypothesis that attention can be shifted to certain depth planes.

In an ERP experiment [12] that investigated a paradigm similar to the one used by Theeuwes et al. [11], the authors subsequently presented their participants bars of different orientation (vertical vs. horizontal) and depth (in front vs. behind the monitor plane). Participants pressed a button upon the detection of the cued target stimulus, which could be any combination of orientation and depth. Target stimuli of the attend-to depth as well as orientation resulted in greater selection negativity (SN), N2b, and P3b amplitudes. The SN component is mostly associated with the processing of a stimulus feature or feature combination, such as color and/or orientation [13,14,15] and is not primarily linked to the processing of spatial information. The P3b is mainly thought of as depicting the updating of context information, i.e., “that is a target stimulus” vs. “that is a distractor stimulus” [16]. Its amplitude reflects the amount of attentional resources allocated to the target stimulus [15]. In a follow-up study [17], Kasai et al. corroborated their own results by manipulating 2D (left vs. right) and 3D position (in front vs. behind the monitor plane) independently. Again, they instructed participants to respond by button presses to one of the targets defined by a specific combination of the two dimensions. Increased amplitudes were found in the P1, N1, and SN components for the to-be-attended-to vs. to-be-ignored 3D position.

Taken together, these results suggest that the SN is a reliable neural marker of differential attentional deployment across depth planes. Both the emergence of SN and P3b indicate the contribution of higher visual areas to this process. However, this provides neither evidence about the temporal dynamics of sustained attentional selection nor about the neural activity during attentional processing of simultaneously presented stimuli.

The steady-state visual evoked potential is predestined to fill these knowledge gaps [18,19,20,21] because it is measured in EEG as the ongoing oscillatory response to a periodically flickering stimulus. By flickering different stimuli consistently at different frequencies, amplitudes of these known frequencies can later be extracted from the combined EEG signal and their dynamics attributed to the processing of the respective stimulus. Thus, frequency-tagging acts as an elegant way to measure cortical activity in simultaneously presented stimuli. Furthermore, due to its focus on the early visual cortex, the SSVEP should depict differences in the activation of 3D representations in early visual areas [22].

Extracted SSVEP amplitudes typically show an increase in amplitude for to-be-attended-to vs. to-be-ignored stimuli [19]. The SSVEP’s suitability to measure temporal neural dynamics has already been proven by several studies that investigated spatial attention in the early visual cortex [20,21,23]. While these experiments provided valuable insights into attentional dynamics, their ability to illuminate the mechanisms of attentional shifts between depth planes remains limited.

In the present study, we measured SSVEPs and ERPs concurrently in a 3D attention paradigm to examine the attentional dynamics that are involved in the early visual processing of different depth stimuli. In doing so, the ecological validity of the results should be improved since attentional shifts in everyday life very likely occur not just in two dimensions but also in spatial depth [24].

To this end, we showed our participants two centrally presented random-dot kinematograms (RDKs) flickering at different frequencies to evoke distinguishable SSVEPs. The displacement of both RDKs in depth was achieved by a stereoscopic 3D setup using circularly polarized stimuli in binocular disparity combined with passive glasses. While participants fixated on a central cross, after a baseline period, they were cued by a color cue to shift attention to one of the planes and to detect and respond to coherent motion events in the to-be-attended-to RDK while ignoring such events in the to-be-ignored plane. With this design, we were able to concurrently analyze the time course of SSVEP amplitudes after the shifting cue and ERPs that were elicited by the coherent motion events [25]. Based on our previous SSVEP studies reported above, we expected an increase in SSVEP amplitudes, relative to the pre-cue baseline for the to-be-attended-to plane/RDK. In our previous feature-based studies, in which we presented two spatially superimposed RDKs with two different colors [26,27], we consistently found a suppression of the to-be-ignored RDK, relative to the pre-cue baseline. Given these findings, we also expected a suppression of the to-be-ignored plane/RDK. With respect to the ERPs elicited by the coherent motion events, we hypothesized greater SN amplitudes for the attended-to than the unattended-to depth plane, as suggested by the studies outlined above. Further, we also expected to find greater P3b amplitudes for coherent motion events in the to-be-attended-to compared to the to-be-ignored plane/RDK [23].

While we were able to replicate SN and P3b amplitude differences [12,17], to our surprise, SSVEP amplitudes did not differ as a function of attentional deployment to either plane/RDK.

## 2. Materials and Methods

### 2.1. Sample

Twenty-six participants were recruited, from which 23 remained in the sample (19 female, mean age = 24.0 years, SD = 4.2, range 18.0–33.0 years; 3 left-handed; 12 with normal, 11 with corrected-to-normal visual acuity). Three participants were excluded, one due to not perceiving stereoscopic depth and two due to excessive EEG artifacts. All participants gave informed consent, and the experiment followed the Declaration of Helsinki and was approved by the local ethics committee (protocol code 298/17-ek, September 2017).

### 2.2. Stimulation Setup

Two overlapping circular random-dot kinematograms (RDKs) with a diameter of 6.92° of visual angle (va) at a viewing distance of 120 cm were presented centrally on the projection screen, each comprising 80 solid white (RGB 1, 1, 1, 1; luminance 100.415 cd/m^2^) squared dots (edge length 0.334° va) moving randomly and independently at a speed of 3.80° va per second. The mutual occlusion of single dots was randomized across presentation frames to prevent local depth cues. Distinct coherent motion events were realized by moving 32 dots of one RDK coherently in one cardinal direction over a period of 300 ms, resulting in a shift of 1.14° va. A fixation cross (line length 0.382° va, line width 0.072° va) was presented centrally in white (RGB 1, 1, 1, 1; luminance 100.415 cd/m^2^), orange (RGB 1, 0.4, 0, 1), or blue (RGB 0, 0.4, 1, 1) colors. All stimuli were shown against a dark gray (RGB 0.05, 0.05, 0.05, 1; luminance 0.041 cd/m^2^) background.

The 3D setup was realized using a polarized projector beam (VPixx Technologies Inc., Montreal, QC, Canada, 2020) with circular polarizer switching every 50 μs and passive 3D glasses. The projector ran at a refresh rate of 480 Hz, and every other image was polarized either clockwise or counter-clockwise. To ensure a consistent positioning of the depth planes across participants, the individual pupil–nose distance was measured. The mean pupil–nose distance of the sample was 31.9 mm (SD = 1.5 mm, range 28.0–34.0 mm). The fixation cross was presented in the plane of fixation, while both RDKs were in front and behind it (distance to the plane of fixation was 10 cm in uncrossed and crossed setups). This setup of symmetric disparities was chosen to prevent participants from solving the task by merely judging differences in the respective disparities. The average of the individually calculated absolute disparity was 0.25° va (SD = 0.01°, range 0.23–0.27°).

Each RDK flickered in on–off cycles at a specific frequency that was calculated using a temporal frame interpolation method [28]. One RDK flickered at 15 Hz, and the other flickered at 18 Hz, while the respective frequencies were counterbalanced across subjects to control the mapping of flicker frequencies to depth planes. These frequencies were used because they provide a sufficient signal-to-noise ratio while still avoiding both the alpha-band and fusion frequency ranges, as observed previously [20].

### 2.3. Task

**Figure 1:**
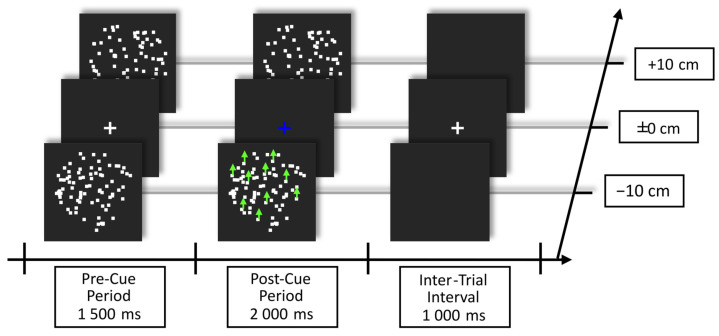
Example of trial structure showing the blue cue instructing the participants to shift attention to the RDK in front (an orange cue would instruct to shift attention to the RDK behind the fixation plane). The color of shift instruction was counterbalanced across subjects. Green arrows depict the coherent motion event that 20 out of 80 dots in one RDK performed during the post-cue period. Note that Brownian motion occurred in both RDKs during the pre- and post-cue period. Participants were instructed to maintain fixation on the cross during the whole experiment and to react to coherent motion events only in the cued RDK.

Trials started with a pre-cue period, in which both RDKs were presented in Brownian motion with the fixation cross in the center against a dark gray background. After this period, the fixation cross changed color in a trial-by-trial manner to either blue or orange, informing the participant to shift attention to one of the two planes/RDKs. The meaning of the color cue was counterbalanced between participants. Starting 200 ms after cue onset, coherent motion events could occur at random time points in any one of the RDKs. The delay between coherent motion events was at least 800 ms. The post-cue period lasted for 2000 ms and was followed by an inter-trial interval of 1000 ms, during which only the fixation cross was presented. The trial structure is shown in Figure 1.

Participants attended to the RDK behind or in front of the fixation cross, depending on the cue color, while maintaining their gaze at the fixation cross. They reacted to coherent motion events in the to-be-attended-to plane/RDK by pressing the space bar of a standard QWERTZ keyboard while ignoring all such events in the other plane/RDK. Hand usage was counterbalanced among participants; i.e., after 50% of the experiment, the reaction was switched to the other hand. Participants were instructed to respond as accurately and as fast as possible.

The experiment included 960 trials in total, presented in 8 blocks. Of these, 480 trials included no coherent motion events, 240 trials included one event, and 240 included two events. After each block, participants received feedback on their performance. The next block was started by the participant by pressing the spacebar. Before the experiment, participants underwent a short 3D exposure and task training consisting of at least 48 trials (until they scored at least 80% hits and fewer than 20% false alarms).

### 2.4. EEG Recording and Processing

EEG was recorded from 64 Ag/AgCl electrodes mounted in an elastic cap with an ActiveTwo Amplifier (BioSemi, Amsterdam, The Netherlands) at a sampling rate of 512 Hz with a low-pass filter of 102.4 Hz and stored for later offline analysis. Bipolar vertical and horizontal electrooculogram montages were utilized to record blinks and horizontal eye movements. For all EEG analyses, EEGLAB [29] was used in combination with in-house MATLAB (Version 24.1.0.2689473 (R2024a)) scripts [30] that followed our lab standard.

For all EEG analyses, the DC offset was subtracted from the continuous EEG. Preprocessing of the SSVEPs following this step differed from the preprocessing for ERPs in the way data were filtered and epoched, as described below. However, for both signals, the respective epochs that contained blinks were discarded based on an in-house adaptive threshold procedure. Epochs that contained lateral eye movements were discarded likewise, with lateral eye movements being defined as saccades larger than 2° va, detected by bipolar 30 µV increases in electrooculogram amplitude. Given this, on average, 8.4% (SD = 7.6%, range 1.5–38.1%) of epochs were discarded per participant for the SSVEP and 5.7% (SD = 3.6%, range 0.3–14.7%) for the ERP analysis. Using the “Statistical control of artifacts in dense array EEG/MEG studies” [31], epochs were scanned for contaminated electrode channels. All epochs that showed more than 12 contaminated channels were discarded from further analysis. For epochs with fewer contaminated channels, these channels were interpolated by weighted spherical splines. On average, 13.9% (SD = 8.5%, range 4.0–34.8%) of epochs were discarded per participant for the SSVEP analysis, and 6.7% (SD = 4.4%, range 0.8–19.4%) for the ERP analysis because of artifact contamination. After this, data were transformed to the average reference. The surface Laplacian was computed to estimate the current source density [32,33].

#### 2.4.1. SSVEP Preprocessing

Epochs were extracted from −1000 ms to +2000 ms relative to cue onset from trials that contained no coherent motion events. Epochs were detrended and averaged for each attentional condition (attended-to/unattended-to plane/RDK) and participant across the respective frequencies.

**Figure 2:**
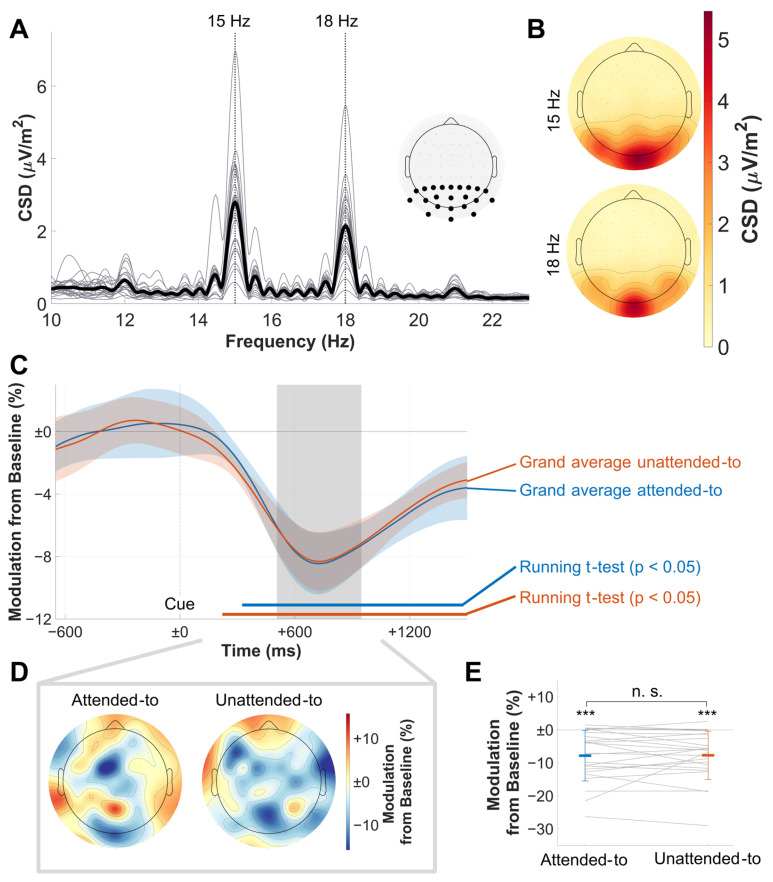
Results of the SSVEP analysis. (**A**) Single-subject (thin lines) and grand average (bold line) spectrograms of the pre-cue period (CSD values) and the electrode cluster used to further analyze data. Note the prominent peaks at the driving flicker frequencies. (**B**) Grand mean SSVEP topographies of the respective flicker frequencies. Note the central occipital activation pattern. (**C**) Baseline-normalized SSVEP amplitude time courses collapsed across frequencies for attentional conditions. Confidence intervals for comparisons between the conditions are depicted as shaded areas. FDR-corrected running *t*-tests for the comparison of each condition against baseline are shown in bold horizontal lines. Note the identical decrease of amplitudes in both to-be-attended-to and to-be-ignored RDKs. The area shaded in gray depicts the chosen time window for further analysis, which was centered around the trough of both time courses. Cue onset at time point zero. (**D**) Topographies for extracted amplitudes in the chosen time window, depicted as a percentage in CSD value modulation from baseline. In contrast to (**B**), there is no clear central activation pattern here. (**E**) Comparison of the extracted mean amplitudes from (**C**), with bold-colored horizontal bars showing mean amplitudes for each condition and thin colored lines showing the respective standard deviations. Gray lines show single-subject data; significant differences are denoted for *t*-test comparisons to baseline and between conditions. Note that there is a highly significant decrease in both conditions below baseline, but no significant difference between the conditions. Colormaps used were provided by the ColorBrewer MATLAB package [36]. n. s. not significant, *** *p* < 0.001.

To determine relevant electrode clusters for the Gabor-filtered time courses of the full SSVEP epochs, SSVEP spectra were calculated for the pre-cue period using Fast Fourier transformation (zero-padding to 2^14^ data points) and collapsed across all experimental conditions. The pre-cue period was chosen to allow for an analysis based on the pure SSVEP signal only, without modulation by attentional shifts. The extracted amplitudes at the respective driving frequencies (15 Hz and 18 Hz, respectively) showed a central occipital activation pattern for both frequencies (see Figure 2B). We decided to use a large electrode cluster for statistical analysis (P9, P7, P5, P3, P1, Pz, P2, P4, P6, P8, P10, PO7, PO3, POz, PO4, PO8, O1, Oz, O2, I1, Iz, and I2) to incorporate interindividual differences in the topographies of peak amplitudes.

SSVEP epochs, containing both the pre- and post-cue periods, were transformed to the frequency domain with a frequency-specific Gabor filter [26] centered at either 15 or 18 Hz (FWHM_frequency_ = ±1 Hz, FWHM_time_ = ±221 ms) to extract the SSVEP time courses, respectively. This relatively wide FWHM in the time domain was needed to attenuate cross-frequency contamination/smearing of the amplitude time courses. Time courses were baseline-normalized by calculating the percentage change to baseline for each data point. The baseline window for all experimental conditions was between −600 to −230 ms relative to cue onset, and the average was calculated. The baseline was chosen to maintain a distance of at least one filter width from the cue onset and to avoid contamination of the onset ERP at the beginning of a trial.

#### 2.4.2. ERP Preprocessing

**Figure 3:**
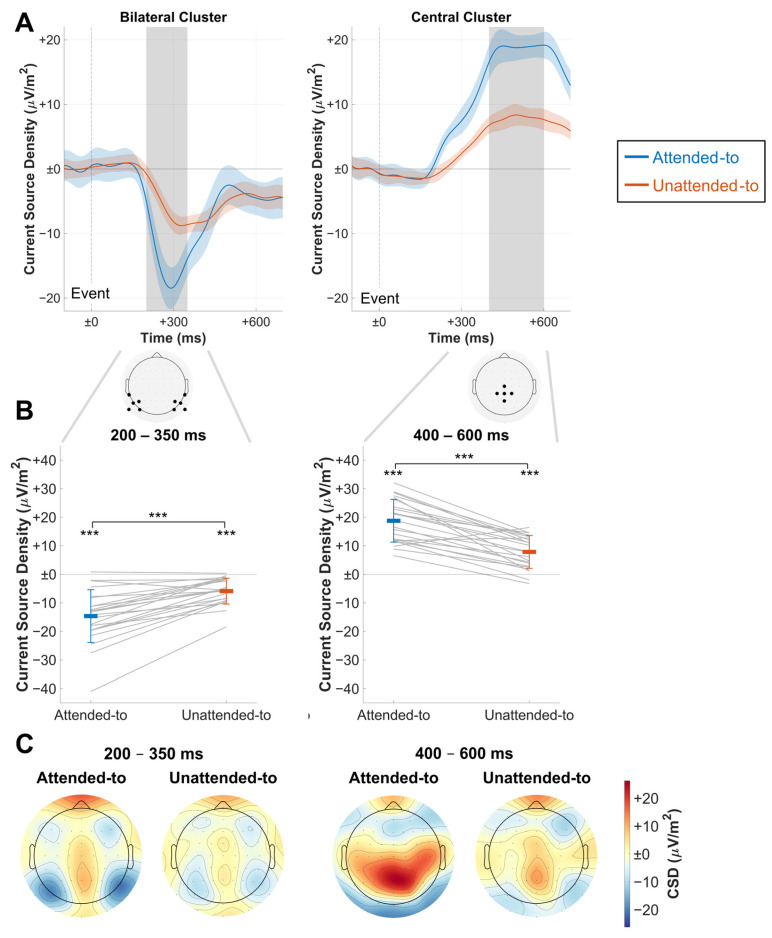
Results of ERP analysis. (**A**) Grand average ERP time courses for an average of the central and bilateral electrode cluster in CSD value differences to baseline (−100 ms to event onset at time point zero). Confidence intervals for comparisons between the conditions are depicted as shaded areas. Electrodes of the respective clusters are shown below. Gray areas depict the time windows used to extract amplitudes for the subsequent figures, which were centered around the visible peaks. Note the clear differences in the respective components. (**B**) Time window averages of both clusters, extracted from (**A**) and depicted as CSD value differences to baseline. Bold-colored horizontal bars depict mean amplitudes for each condition, and thin colored lines depict the respective standard deviations. Gray lines show single-subject data; significant differences are denoted for *t*-test comparisons against the baseline and between conditions. (**C**) ERP activity as CSD value differences to baseline during the time windows defined in (**A**). Note the bilateral parieto-occipital activation pattern during the early time window and the central parietal activity peak during the late time window. Colormaps used were provided by the ColorBrewer MATLAB package [36]. *** *p* < 0.001.

For ERP analysis, continuous data were filtered with a low-pass filter at 11.5 Hz to mitigate biases from SSVEP on the ERP. After that, epochs were extracted from −100 ms to 700 ms relative to coherent-motion event onset. Only events with correct behavioral responses were considered for ERP analysis. Two different electrode clusters were extracted from both to-be-attended-to and to-be-ignored event epochs, an occipital bilateral cluster (TP7, P9, P7, P5, PO7, TP8, P6, P8, P10, and PO8) and a parieto-central cluster (Cz, CP1, CPz, CP2, and Pz, Figure 3A). This decision was made because the SN was expected to be most pronounced in occipital regions [13,14], while the P3b was expected to be elicited in central parietal regions [16]. Electrode channels were averaged for both clusters. ERPs were baseline-corrected by subtracting the mean of the baseline from all data points, which was chosen to range from −100 ms to event onset.

### 2.5. Statistical Analysis

#### 2.5.1. Behavioral Analysis

For behavioral analyses, in-house MATLAB scripts or R (v4.4.1; R Core Team 2024) [34] scripts were used. Button presses made within 200 to 1000 ms after a coherent motion event onset in the to-be-attended-to plane/RDK were considered hits. Responses to coherent motion events in the to-be-ignored plane/RDK were considered false alarms. Hit rates were calculated as the proportion of the actual number of hits to the maximum possible number of hits. False-alarm rates were calculated in the same manner. The sensitivity index d’ was calculated by using a correction for extreme hit and false-alarm rates [35].

#### 2.5.2. SSVEP Analysis

Baseline-normalized time courses were averaged across all electrodes that were previously determined to be within the relevant electrode cluster. Resulting data were collapsed to depict the amplitude modulations of the to-be-attended-to vs. to-be-ignored RDKs/planes after a preliminary analysis showed similar time courses for foreground and background RDKs.

SSVEP time courses were subjected to a running FDR-corrected two-tailed *t*-test for paired samples (*p* < 0.05) [37,38]. This was done to (a) determine the windows at which SSVEP amplitude modulations differed significantly from baseline and to (b) test SSVEP time courses between the to-be-attended-to and to-be-ignored planes/RDKs. Confidence intervals used to visualize significant differences between the time courses were calculated using the approach of Morey et al. [39,40]. For further statistical testing, we employed post-hoc analyses with the mean amplitude of a time window ranging from 506 ms to 948 ms, during which SSVEP amplitude reductions were clearly established. To test for possible differences between conditions in this time window, we used a two-tailed paired-samples Bayesian [41] and frequentist *t*-test.

#### 2.5.3. ERP Analysis

The baseline-corrected ERPs for both to-be-attended-to and to-be-ignored events were averaged separately. Confidence intervals used to visualize significant differences between the time courses were again calculated using the Morey et al. method [39,40]. The SN component was extracted by averaging data points in the bilateral cluster between 200 and 350 ms. For the P3b component, data points in the central cluster were extracted between 400 and 600 ms and averaged. Comparisons for the extracted amplitudes were obtained by calculating a two-tailed paired-samples Bayesian and frequentist *t*-test for each component (Figure 3B).

#### 2.5.4. Control Experiment Analysis

A control experiment tested the hypothesis that participants used the flicker frequency instead of the depth cue to solve the experimental task. To this end, in two blocks, participants performed the task either with identical stimulation as in the EEG experiment, i.e., with RDKs flickering at different frequencies, or with both RDKs flickering at the same frequency. Each block consisted of 480 trials which always contained events, 240 containing one event and 240 containing two events. The order of blocks was counterbalanced across participants.

For this control experiment, nine participants were recruited. One participant had to be excluded due to performance at chance level, leaving eight participants (seven female, one diverse, mean age = 27.6 years, SD = 7.2, range 21.0–44.0 years). Most of them were right-handed (2 were left-handed), six had normal visual acuity, and two had corrected-to-normal visual acuity.

Behavioral measures were used to compare the performance of the participants between different-frequency and same-frequency blocks. This test was important to exclude the possibility that subjects used frequency rather than depth information to discriminate between and attentionally select the two RDKs. If that were the case, we would expect much higher false-alarm rates and a drop in performance when subjects performed the task when both RDKs flickered at the same frequency. To this end, reaction times, hit and false-alarm rates, and the sensitivity index d’ were compared. For all comparisons, Wilcoxon signed-rank tests were employed to account for the small sample size. To allow for meaningful inferences about the null hypothesis, Wilcoxon signed-rank test statistics were also analyzed using a Bayesian approach [42].

## 3. Results

Bayes factors are always reported as depicting evidence for the alternative hypothesis and interpreted using guidelines by Kass and Raftery [43].

### 3.1. Behavioral Data

Results indicated that the task was demanding participants performed well and were able to solve the task (reaction time: mean = 549.729 ms, SD = 49.230 ms; hit rates: mean = 92.650%, SD = 5.530%; false-alarm rates: mean = 3.998%, SD = 4.216%; d’: mean = 2.718, SD = 0.437). Thus, they were able to separate the depth planes; otherwise, behavioral performance would have been at the chance level.

### 3.2. SSVEP Data

Both stimulation frequencies elicited pronounced SSVEPs (Figure 2A), with a prominent maximum at central occipital electrodes in the grand mean topographical distribution (Figure 2B). As depicted in Figure 2C, SSVEP amplitude time courses showed significant deviations from baseline with a pronounced negative deflection after cue onset for both the to-be-attended-to (320 ms to trial end) and to-be-ignored planes/RDKs (219 ms to trial end). However, the time courses were not significantly different from each other (*p* > 0.1 for every time point). The *t*-test of the time window for a comparison of the attentional conditions did not yield any significant results either (t(22) = −0.114, *p* = 0.911, d = −0.024), and Bayesian statistics found positive evidence for the null hypothesis (BF10 = 0.220, Figure 2E).

### 3.3. ERP Data

As depicted in Figure 3A, ERPs elicited by coherent motion events showed a pronounced negative deviation from baseline at approximately 200 ms until approximately 500 ms for both to-be-attended-to and to-be-ignored events. This deflection formed a bilateral parieto-occipital activation pattern (Figure 3C). The extracted amplitudes of this component showed CSD values to be higher for to-be-attended-to than to-be-ignored events between 200 and 350 ms (Figure 3B). This was confirmed in a frequentist analysis that showed a highly significant difference (t(22) = −6.488, *p* < 0.001, d = −1.353) and a Bayesian analysis that found very strong evidence for the alternative hypothesis (BF10 = 11,753.764).

Also, a pronounced positive deflection was elicited, starting to deviate from the baseline at about 230 ms and lasting until the end of the epoch (Figure 3A). This positive component was observed at central electrodes, stretching from central to parieto-occipital regions (Figure 3C). Amplitudes extracted between 400 and 600 ms showed a greater amplitude for to-be-attended-to than for to-be-ignored events (Figure 3B). Again, a frequentist *t*-test found a significant difference (t(22) = 7.710, *p* < 0.001, d = 1.608), and a Bayesian *t*-test found very strong evidence for the alternative hypothesis (BF10 = 139,593.033).

### 3.4. Control Experiment

On average, participants in different-frequency trials achieved reaction times of 498 ms (SD = 39 ms) and 506 ms (SD = 40 ms) in same-frequency trials, which did not result in a significant difference (z = −0.105, *p* = 0.917) in the frequentist analysis, while a Bayesian analysis found positive evidence for the null hypothesis (BF10 = 0.302). In different-frequency trials, they had hit rates of 97.0% (SD = 3.2%) on average, while they averaged 97.5% (SD = 2.8%) in same-frequency trials, which again resulted in no significant differences (z = −1.82, *p* = 0.069) and strong evidence for the null hypothesis (BF10 = 0.048) in the Bayesian analysis. False-alarm rates averaged 2.8% (SD = 3.0%) in different-frequency trials and 3.5% (SD = 2.0%) in same-frequency trials, showing no significant difference (z = −1.12, *p* = 0.263), while Bayesian statistics provided positive evidence for the null hypothesis (BF10 = 0.219). Consequently, the sensitivity index was calculated for different-frequency trials (d’ mean = 3.42, SD = 0.51) and same-frequency trials (d’ mean = 3.36, SD = 0.41), respectively, resulting in a non-significant difference (z = 0.700, *p* = 0.484) but indecisive evidence in the Bayesian test (BF10 = 2.988).

## 4. Discussion

In the present study, we aimed to examine electrophysiological measurements in order to uncover the neural mechanisms underlying depth perception. We explored a novel experimental 3D setup by presenting two RDKs that flickered at two different frequencies and were located on different stereoscopic depth planes. This allowed for the concurrent analysis of continuous RDK-driven SSVEPs and ERPs that were elicited by to-be-detected coherent motion events. In particular, we were interested in uncovering the temporal neural dynamics of attentional shifts in 3D space.

We were able to replicate previous electrophysiological findings of enlarged amplitudes for the SN and P3b for coherent motion events in the to-be-attended-to compared to the to-be-ignored plane [12,17]. Although our novel experimental setup evoked reliable SSVEPs, surprisingly to us, after the presentation of the attentional shifting cue, SSVEP amplitude modulations did not differ between the two planes/RDKs and exhibited a pronounced amplitude reduction compared to the pre-cue baseline period. Importantly, hit rates of about 93% in combination with a very low false-alarm rate of only about 4% clearly demonstrated that subjects were able to perform the task, i.e., were able to discriminate between the two planes/RDKs. In a control experiment, we could also rule out that participants selected the cued RDKs based on their frequencies to solve the behavioral task instead of selecting them based on their depth. Participants performed just as well when RDKs flickered at identical or different temporal frequencies, resembling the findings of previous control analyses [19]. This again strengthens the internal validity of the RDK paradigm by showing that flicker frequency is not used as an additional aiding cue to discriminate and attentionally select the RDKs and thus solve the task. In light of the other results in the current experiment, it provides evidence for a disparity-based RDK selection mechanism.

The visual stimulation was clearly able to evoke SSVEPs distinguishable in the frequency domain, displaying an expected topographic distribution over occipital electrodes. Yet, no selective attentional modulation of these signals was measurable. In our previous SSVEP studies investigating attention in 2D space, however, we consistently found a significant SSVEP amplitude enhancement after cue onset for the to-be-attended-to location relative to the pre-cue baseline [18,20]. Utilizing a design similar to the present one, our previous SSVEP studies measured neural temporal dynamics in feature-based attention by using spatially superimposed but differently colored RDKs within a single depth plane. When we cued subjects to shift attention to one of the two colors, we consistently found SSVEP amplitude amplification for the to-be-attended-to color and SSVEP amplitude suppression for the to-be-ignored color relative to the pre-cue baseline [26,27]. In previous experiments investigating spatial shifts of attention within 2D space, i.e., the fixation plane, no suppression was found for the to-be-ignored stimulus position, while an early amplification was still observed [18,20]. To-be-ignored stimuli were most likely not suppressed because of their spatial separation. This prevented direct competition for processing resources [44]. Given these consistent patterns in previous results, we expected a pattern of amplification and suppression in the present study when subjects shifted attention to one of the planes/RDKs after the cue.

Why are SSVEP amplitude responses indicative of attentional selection in 2D environments but not in 3D environments? Single-cell studies mapping visual processing areas in stereoscopic depth perception might resolve this question.

According to single-cell studies that manipulated the retinal input to both eyes independently in macaques [6], binocular cells in area V1 responded equally to retinal input, resulting in stimuli perceived in front of the plane of fixation and behind the plane of fixation. In other words, they do not solve the stereoscopic correspondence problem because they are firing indiscriminately in response to both correct and incorrect matches. Accordingly, binocular neurons in V1 are not sensitive to the phenomenological perception of depth in a stimulus display. Only at later stages of the ventral visual processing pathway, in the anterior part of the inferior temporal cortex, is neuronal activity strictly tuned to retinal input associated with a meaningful depth perception and even correlated with the phenomenological depth perception of the monkeys [45]. These results suggest that at some point between area V1 and the anterior inferior temporal cortex, global depth perception is solved [46]. Stereoscopic vision, in general, seems to emerge as a consequence of neural processing along the ventral and dorsal pathway [4,5], and SSVEPs, which reportedly originate in the early visual cortex [18], may not allow for depth-selective processing and attentional selection thereof.

Taking into consideration that the SSVEP response to RDKs centered around the point of fixation is most pronounced in early visual processing areas, this may explain why SSVEP amplitude time courses in the present experiment did not differ. Although the presentation of overlapping flickering stimuli leads to neural activation in those early visual processing areas that we can measure as SSVEPs, their time courses do not differ between attentional conditions because binocular neurons may respond to all stimuli at a certain 2D position in visual space, irrespective of the different distances from the plane of fixation. Neuron populations in early visual areas driving the SSVEP signal may not differentiate between stimuli that are spatially overlapping but different in depth and may thus not be gain-modulated by attentional selection in depth.

Contrasting this, the emergent SN and P3b components appear to provide a more robust explanation of behavioral performance during a stereoscopic selection task. This is in line with the explanation provided above: since the generating sources of both components are not situated in the early visual cortex, they might be able to depict attentional shifts between depth planes better than SSVEPs.

The observed selective modulation of the ERP resembles the topographical and temporal dynamics of the Selection Negativity (SN) component. The SN component is described as a difference potential in the ERP when subjects either attended to a certain feature of a stimulus (such as a certain color) or when this feature was unattended to, or when a certain feature was present compared to the absence of such a feature [13]. It begins after approximately 150 ms and lasts for 200 ms, with activity peaking in posterior regions [47]. Anllo-Vento et al. [14] introduced a typical experimental setup to evoke the SN by serially presenting blue or red checkerboard patterns to participants and instructing them before each experimental block to react to slight luminance changes in one of the two colors. They found a prominent SN distributed over the posterior visual cortex when comparing ERPs elicited by the to-be-attended-to and the to-be-ignored colors. Crucially, in these studies, the SN was not selective to the spatial position of the stimuli, which led to the interpretation of the SN as a marker for differences in feature-based attentional resource distribution.

The SN is generally considered to be a signature of activity in brain areas related to the neural processing of attentionally selected stimuli [48]. For spatially overlapping stimuli of different colors, the generators of the SN for the attended-to over the unattended-to stimulus are found in areas such as V4 [14,49]. Crucially, previous studies found an SN component for overlapping stimuli that differed in stereoscopic depth and for which one of these stimuli was cued and attentionally selected while the other one was unattended to [12,17].

Later, a positivity for task-relevant events in the cued and attended-to depth plane over unattended-to events arises over parietal regions that may resemble a P3b component [16]. While no scientific consensus about the exact generators has yet been reached, its signal seems to be produced by connectional activity between frontal and temporal/parietal areas in the brain and, thus, is higher up in the processing hierarchy. As this component was found to be related to awareness processes and response preparation for task-relevant and attended-to stimuli [16,47,50,51], it was often observable in our own previous studies into spatially overlapping stimuli that were selected and attended to based on their color [52,53].

Taken together, these ERP components suggest the source of the signal that allocates attentional resources between depth planes is situated beyond early visual processing areas. This further corroborates the notion that early visual processing does not mainly contribute to the differentiation of disparities and the allocation of attention during the reported task.

If SSVEP amplitudes may not be indicative of depth-selective attention, what is the reason for the non-selective SSVEP amplitude drop that begins approximately 250 ms after cue onset? In earlier works, amplitude drops after cue onset were consistently found for to-be-ignored stimuli following the amplification of the to-be-attended-to stimulus. Thus, it was hypothesized that this delayed suppression of stimuli is driven by a feedback mechanism that originates in cortical areas representing larger receptive fields and aids attentional stimulus selection in the early visual cortex [26,27].

However, the amplitude decrease found in the current study is not attention-selective, as it depicts comparable modulations for the cued and uncued RDKs and cannot easily be explained by a mechanism of feedback-triggered, delayed suppression. As SSVEP amplitude modulations have been linked to early sensory gain modulation for stimulus processing in the early visual cortex [25], the amplitude decrease may depict an attenuated stimulus processing following the cue. No such pattern was a priori expected and, thus, warrants further research to be explained.

## 5. Conclusions

We concurrently measured electrophysiological markers of ongoing attentional resources and transient stimulus processing in humans to test both methods in a novel setup evoking stereoscopic 3D perception. While we were able to replicate modulations of the SN and P3b components as a function of attentional deployment to one of the planes/RDKs, surprisingly, SSVEP amplitudes did not differentiate between attentional conditions. Behavioral data from this and a control study showed that participants solved the task at a high performance level. Taken together, these results suggest that the early visual cortex may not optimally represent depth-selective attention while corroborating existing ERP findings. Future research should aim to investigate the role of the SN in the attentional processing of 3D space while being aware of the fact that SSVEPs may not be suited for studying neural temporal dynamics in depth-selective attention.

## Data Availability

The data and scripts used in this study are available from J.J. upon reasonable request.
